# Supplementary material for: Nanoscopic anatomy of dynamic multi-protein complexes at membranes resolved by graphene-induced energy transfer
Source: eLife. 2021 Jan 29;10:e62501. doi: 10.7554/eLife.62501 (PMC7847308; doi:10.7554/eLife.62501)
Supplement: Supplementary file 1. [file elife-62501-supp1.docx]

1. **Table S1 Sequences and modifications of DNA strands**

| DNA Strands | Sequence and modifications (5'-3') ^a^ |
| --- | --- |
| 20mer anchor: | GATGAATGGTGGGTGAGAGG-TEG-Cholesterol |
| 25mer anchor: | GATGAATGGTGGGTGAGAGGTGAGG-TEG-Cholesterol |
| 35mer anchor: | GATGAATGGTGGGTGAGAGGTGAGGAGTAAGAGGA-TEG-Cholesterol |
| 50mer anchor: | GATGAATGGTGGGTGAGAGGTGAGGAGTAAGAGGATGTGTTAGAGGGATG-TEG-Cholesterol |
| 3´-FAM probe: | CCTCTCACCCACCATTCATC-FAM |
| 5´-FAM probe: | FAM-CCTCTCACCCACCATTCATC |
| 15 mer blocker: | TCCTCTTACTCCTCA |
| 30 mer blocker: | CATCCCTCTAACACATCCTCTTACTCCTCA |

^a^: TEG represents the tri-ethylene glycol phosphate linker.

1. **Table S2 Fluorescence intensity ratios for GIET calibration ^a^**

| Rulers **^b^** | Intensity on graphene (mV) | Intensity on silica (mV) | Intensity ratio**^c^** (%) | *d* (nm)**^d^** |
| --- | --- | --- | --- | --- |
| OG488-DHPE | 5.4 ± 0.4 | 322.1 ± 4.0 | 3.8 ± 0.2 | 2.5 |
| 25-5´F | 9.9 ± 0.5 | 138.3 ± 1.2 | 7.2 ± 0.3 | 3.6 |
| 35-5´F | 69.6 ± 1.9 | 550.5 ± 1.2 | 12.6 ± 0.4 | 5.9 |
| 20-3´F | 20.7 ± 0.5 | 116.5 ± 1.2 | 17.8 ± 0.4 | 7.0 |
| 25-3´F | 13.7 ± 0.6 | 59.3 ± 2.2 | 23.2 ± 1.4 | 8.1 |
| 50-5´F | 137.4 ± 0.7 | 328.2 ± 1.6 | 41.9 ± 0.3 | 9.3 |
| 35-3´F | 35.7 ± 0.7 | 75.2 ± 1.8 | 47.6 ± 1.5 | 10.4 |
| 50-3´F | 20.4 ± 0.5 | 32.0 ± 0.6 | 63.8 ± 1.9 | 13.8 |

^a^: Data are presented as mean ± s.d. from 3 different acquisition time points after formation of lipid layers or hybridization of DNA strands. ^b^: Hybridized DNA strands are denoted as anchor strand-3´F or 5´F. ^c^: Normalized by mass factor *m_0_/m_G_* using Eqn (1). *m_0_/m_G_* is 1 for DNA strands and is 2.3 for OG488-DHPE as determined by mass signals. ^d^: Vertical distance obtained by global fitting using Eqn (3).

1. **Table S3 Fluorescence lifetime ratios for GIET calibration ^a^**

| Rulers ^b^ | Lifetime on graphene (ns) | Lifetime on  glass (ns) | Lifetime ratio^c^  (%) | *d* (nm) ^d^ |
| --- | --- | --- | --- | --- |
| OG488-DHPE | N.A. | 3.379 ± 0.059 | -- | -- |
| 25-5´F | 0.239 ± 0.004 | 3.299 ± 0.072 | 7.2 ± 0.2 | 3.7 |
| 35-5´F | 0.394 ± 0.014 | 3.270 ± 0.067 | 12.0 ± 0.5 | 6.0 |
| 20-3´F | 0.552 ± 0.018 | 2.771 ± 0.025 | 19.9 ± 0.7 | 7.2 |
| 25-3´F | 0.786 ± 0.016 | 2.644 ± 0.059 | 29.7 ± 0.9 | 8.4 |
| 50-5´F | 1.369 ± 0.031 | 3.267 ± 0.068 | 41.9 ± 1.3 | 9.5 |
| 35-3´F | 1.268 ± 0.064 | 2.705 ± 0.030 | 46.9 ± 2.4 | 10.7 |
| 50-3´F | 1.979 ± 0.092 | 2.826 ± 0.070 | 70.0 ± 3.7 | 14.2 |

^a^: Data are presented as mean ± s.d. from 3 measurements. ^b^: Hybridized DNA strands are denoted as anchor strand-3´F or 5´F. ^c^: Lifetime of OG488-DHPE on graphene is not available (N.A.) due to detection limitation. ^d^: Vertical distance obtained by global fitting using Eqn (3).
