# Supplementary material for: Nanoscopic anatomy of dynamic multi-protein complexes at membranes resolved by graphene-induced energy transfer
Source: eLife. 2021 Jan 29;10:e62501. doi: 10.7554/eLife.62501 (PMC7847308; doi:10.7554/eLife.62501)
Supplement: Supplementary file 2. [file elife-62501-supp2.docx]

# Supplementary file 2

**Table S4 Fluorescence lifetime ratios of endocytic proteins and protein complexes^a^**

| Protein/protein complexes | Lifetime on graphene (ns) | Lifetime on glass (ns) | Lifetime ratio (%) | *h* (nm) ^b^ |
| --- | --- | --- | --- | --- |
| mNeon-pYpt7-GTP | 0.594 ± 0.059 | 2.832 ± 0.062 | 21.0 ± 2.1 | 4.5 ± 0.3 |
| mNeon-pYpt7-GTP + Mon1-Ccz1 | 0.479 ± 0.037 | 2.828 ± 0.023 | 16.9 ± 1.3 | 3.8 ± 0.2 |
| mNeon-pYpt7-GTP + HOPS | 0.720 ± 0.042 | 2.824 ± 0.013 | 25.6 ± 1.5 | 5.2 ± 0.2 |
| pYpt7-GTP + HOPS Vps39-yEGFP | 1.312 ± 0.039 | 2.088 ± 0.022 | 62.8 ± 1.9 | 9.5 ± 0.3 |
| pYpt7-GTP + HOPS Vps11-yEGFP | 1.182 ± 0.042 | 2.079 ± 0.009 | 56.9 ± 2.0 | 8.5 ± 0.3 |
| pYpt7-GTP + HOPS Vps18-yEGFP | 1.502 ± 0.038 | 2.079 ± 0.007 | 72.2 ± 1.8 | 11.5 ± 0.5 |
| pYpt7-GTP + HOPS Vps16-yEGFP | 1.571 ± 0.026 | 2.123 ± 0.010 | 74.0 ± 1.2 | 12.0 ± 0.3 |
| pYpt7-GTP + HOPS Vps33-yEGFP | 1.490 ± 0.091 | 2.075 ± 0.011 | 71.8 ± 4.4 | 11.4 ± 1.1 |

^a^: Data presented as mean ± s.d. from > 5 measurements. ^b^: Vertical distance to lipid monolayer according to distance-dependent GIET curves.
