# Supplementary material for: Nanoscopic anatomy of dynamic multi-protein complexes at membranes resolved by graphene-induced energy transfer
Source: eLife. 2021 Jan 29;10:e62501. doi: 10.7554/eLife.62501 (PMC7847308; doi:10.7554/eLife.62501)
Supplement: Supplementary file 3. [file elife-62501-supp3.docx]

## Supplementary file 3

**A Table S5 Plasmids used in this study**

| Protein | Backbone | Reference |
| --- | --- | --- |
| EGFP, NB-H6 | pET21a | Novagen |
| Ypt7 | pET24b | Cabrera et al., 2014 |
| Ypt7 | pET24d-GST-TEV | Lachmann et al., 2012 |
| mNeon-Ypt7 | pET24d-GST-TEV | This study |
| Gdi1 | pGEX-6P | Thomas et al., 2016 |
| Bet2-Bet4 | pCDF-DUET-1 | Thomas et al., 2016 |
| Mrs6 | pET30 | Gift from K. Alexandrov |

**B Table S6 Yeast strains used in this study**

| Strain | Genotype | Reference |
| --- | --- | --- |
| CUY2470 | MATa *his3∆200 leu2∆0 met15∆0 trp1∆63 ura3∆0 CCZ1::TRP1-GAL1pr MON1::HIS3MX6-GAL1pr*  *CCZ1::TAP-URA3* | Nordmann et al., 2010 |
| CUY2675 | BY4732xBY4727 *VPS41::TRP1-GAL1pr VPS41::TAP-URA3 VPS39::KanMX-GAL1pr VPS33::HIS3-GAL1pr*  *VPS11::HIS3-GAL1Pr VPS16::natNT2-GAL1Pr VPS18::kanMX-GAL1Pr-3HA* | Ostrowicz et al., 2010 |
| CUY4391 | BY4732xBY4727 *VPS41::TRP1-GAL1pr VPS41::TAP-URA3 VPS39::KanMX-GAL1pr VPS39::yEGFP-hphNT1 VPS33::HIS3-GAL1pr VPS11::HIS3-GAL1Pr*  *VPS16::natNT2-GAL1Pr VPS18::kanMX-GAL1Pr-3HA* | Bröcker et al., 2012 |
| CUY4392 | BY4732xBY4727 *VPS41::TRP1-GAL1pr VPS41::TAP-URA3 VPS39::KanMX-GAL1pr VPS33::HIS3-GAL1pr*  *VPS11::HIS3-GAL1Pr VPS11::yEGFP-hphNT1 VPS16::natNT2-GAL1Pr VPS18::kanMX-GAL1Pr-3HA* | Bröcker et al., 2012 |
| CUY4393 | BY4732xBY4727 *VPS41::TRP1-GAL1pr VPS41::TAP-URA3 VPS39::KanMX-GAL1pr VPS33::HIS3-GAL1pr*  *VPS11::HIS3-GAL1Pr VPS16::natNT2-GAL1Pr VPS16::yEGFP-hphNT1 VPS18::kanMX-GAL1Pr-3HA* | Bröcker et al., 2012 |
| CUY4394 | BY4732xBY4727 *VPS41::TRP1-GAL1pr VPS41::TAP-URA3 VPS39::KanMX-GAL1pr VPS33::HIS3-GAL1pr*  *VPS11::HIS3-GAL1Pr VPS16::natNT2-GAL1Pr VPS18::kanMX-GAL1Pr-3HA VPS18::yEGFP-hphNT1* | Bröcker et al., 2012 |
| CUY4395 | BY4732xBY4727 *VPS41::TRP1-GAL1pr VPS41::TAP-URA3 VPS39::KanMX-GAL1pr VPS33::HIS3-GAL1pr*  *VPS33::yEGFP-hphNT1 VPS11::HIS3-GAL1Pr VPS16::natNT2-GAL1Pr VPS18::kanMX-GAL1Pr-3HA* | Bröcker et al., 2012 |
