# Supplementary material for: Nanoscopic anatomy of dynamic multi-protein complexes at membranes resolved by graphene-induced energy transfer
Source: eLife. 2021 Jan 29;10:e62501. doi: 10.7554/eLife.62501 (PMC7847308; doi:10.7554/eLife.62501)
Supplement: Supplementary file 4. [file elife-62501-supp4.docx]

## Supplementary file 4

**Table S7 Conformational states and transition kinetics from smGIET**

| Protein | HOPS Vps33-yEGFP | | | HOPS Vps11-yEGFP | | |
| --- | --- | --- | --- | --- | --- | --- |
| State | L | M | H | L | M | H |
| *I_G_/I*_0_ ^a^ | 0.25 ± 0.06 | 0.41 ± 0.08 | 0.69 ± 0.13 | 0.22 ± 0.05 | 0.36 ± 0.06 | 0.51 ± 0.08 |
| *h* (nm) ^b^ | 4.7  (3.9 – 5.5) | 6.8  (5.7 - 7.9) | 11.2  (8.9 – 14.9) | 4.3  (3.6 – 5.0) | 6.1  (5.3 - 6.9) | 8.1  (7.0 – 9.3) |
| *Occup.*(%)^c^ | 30.9 ± 0.1 | 35.7 ± 0.2 | 33.5 ± 0.1 | 40.4 ± 0.2 | 38.0 ± 0.2 | 21.6 ± 0.1 |
| Transition counts and rate ***k*** (s^-1^)^d^ | ***k_L-M_***: (471)  1.05 ± 0.05  ***k_L-H_***: (65)  0.15 ± 0.01 | ***k_M-L_***: (470)  0.48 ± 0.02  ***k_M-H_***: (527)  0.54 ± 0.02 | ***k_H-L_***: (77)  0.13 ± 0.01  ***k_H-M_***: (534)  0.87 ± 0.04 | ***k_L-M_***: (398)  0.76 ± 0.04  ***k_L-H_***: (28)  0.05 ± 0.01 | ***k_M-L_***: (392)  0.53 ± 0.02  ***k_M-H_***: (267)  0.36 ± 0.02 | ***k_H-L_***: (34)  0.10 ± 0.01  ***k_H-M_***: (262)  0.73 ± 0.04 |
| *ΔG (k_B_T)^e^* | +0.78 | 0 | +0.48 | +0.36 | 0 | +0.71 |

*^a^*: mean ± s.d. based on *I_G_* of the Gaussian fits in single molecule intensity distribution on graphene. *I*_0_ is the mean value of Gaussian fit on glass (Fig S15).*^b^*: *h* is the height of NB-labeled HOPS on the membrane. Values in brackets are the boundary of *h*. *^c^*: mean ± s.e.m. of state occupancy based on observations of N = 101615 for Vps33, N = 84218 for Vps11, respectively. *^d^*: Transition counts are shown in brackets. Transition rates are presented as mean ± s.e.m. *^e^*: Free energy between the states calculated by $\Delta G=-k_{B}T\ln K$, in which $k_{B}$ is the Boltzmann constant. *T* is the absolute temperature. *K* is the equilibrium constant calculated as the ratio of forward and backward transition rates between M and the target state.
