# Supplementary material for: Nanoscopic anatomy of dynamic multi-protein complexes at membranes resolved by graphene-induced energy transfer
Source: eLife. 2021 Jan 29;10:e62501. doi: 10.7554/eLife.62501 (PMC7847308; doi:10.7554/eLife.62501)
Supplement: Supplementary file 5. [file elife-62501-supp5.docx]

## Supplementary file 5

**Table S8 RMSD and mean intensities of single molecule detections**

| Mean Intensity (a.u.) | 314.1 | 411.1 | 527.4 | 655.7 | 668.3 |
| --- | --- | --- | --- | --- | --- |
| RMSD (a.u.) | 26.2 | 32.2 | 37.1 | 46.4 | 51.1 |
| RMSD/Mean (%) | 8.3 | 7.7 | 7.0 | 7.1 | 7.6 |
| Average (%) | 7.5 ± 0.5 | | | | |

Mean and RMSD were calculated from single molecule intensity traces longer than 100 frames (3.2 s).
